# The NOTCH signaling pathway in normal and malignant blood cell production

**DOI:** 10.1007/s12079-015-0271-0

**Published:** 2015-02-26

**Authors:** Sukanya Suresh, Alexandra E. Irvine

**Affiliations:** 1 Molecular Medicine Branch, National Institute of Diabetes and Digestive and Kidney Diseases, National Institutes of Health, Bethesda, MD 20892 USA; 2 Centre for Cancer Research and Cell Biology, Queen’s University Belfast, Belfast, BT9 7BL N. Ireland UK

**Keywords:** Notch, Haematopoiesis, Leukaemia

## Abstract

The NOTCH pathway is an evolutionarily conserved signalling network, which is fundamental in regulating developmental processes in invertebrates and vertebrates (Gazave et al. in BMC Evol Biol 9:249, 2009). It regulates self-renewal (Butler et al. in Cell Stem Cell 6:251–264, 2010), differentiation (Auderset et al. in Curr Top Microbiol Immunol 360:115–134, 2012), proliferation (VanDussen et al. in Development 139:488–497, 2012) and apoptosis (Cao et al. in APMIS 120:441–450, 2012) of diverse cell types at various stages of their development. NOTCH signalling governs cell-cell interactions and the outcome of such responses is highly context specific. This makes it impossible to generalize about NOTCH functions as it stimulates survival and differentiation of certain cell types, whereas inhibiting these processes in others (Meier-Stiegen et al. in PLoS One 5:e11481, 2010). NOTCH was first identified in 1914 in *Drosophila* and was named after the indentations (notches) present in the wings of the mutant flies (Bigas et al. in Int J Dev Biol 54:1175–1188, 2010). Homologs of NOTCH in vertebrates were initially identified in *Xenopus* (Coffman et al. in Science 249:1438–1441, 1990) and in humans NOTCH was first identified in T-Acute Lymphoblastic Leukaemia (T-ALL) (Ellisen et al. in Cell 66:649–61, 1991). NOTCH signalling is integral in neurogenesis (Mead and Yutzey in Dev Dyn 241:376–389, 2012), myogenesis (Schuster-Gossler et al. in Proc Natl Acad Sci U S A 104:537–542, 2007), haematopoiesis (Bigas et al. in Int J Dev Biol 54:1175–1188, 2010), oogenesis (Xu and Gridley in Genet Res Int 2012:648207, 2012), differentiation of intestinal cells (Okamoto et al. in Am J Physiol Gastrointest Liver Physiol 296:G23–35, 2009) and pancreatic cells (Apelqvist et al. in Nature 400:877–881, 1999). The current review will focus on NOTCH signalling in normal and malignant blood cell production or haematopoiesis.

## NOTCH receptors and ligands

**Fig. 1:**
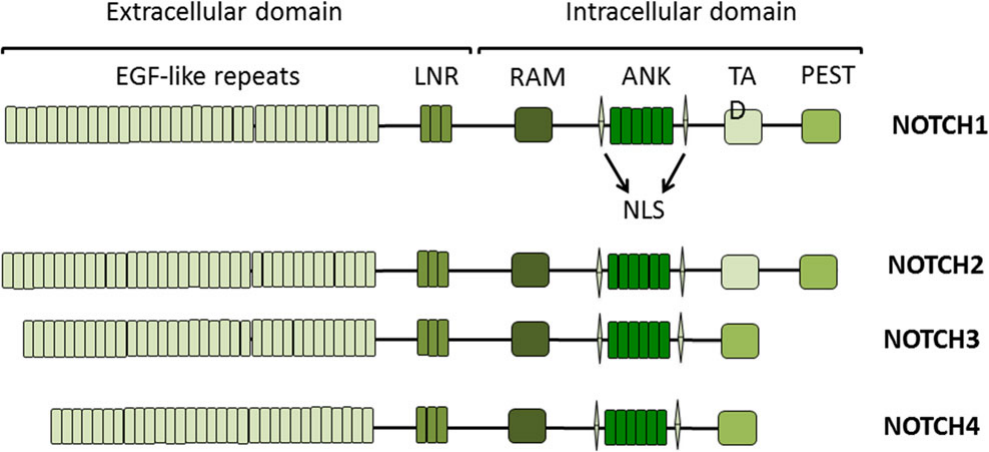
*NOTCH receptors*. The mammalian NOTCH family has four members NOTCH1-4; all consist of extracellular and intracellular domains. The extracellular domain 29–36 Epidermal growth factor (EGF) like repeats followed by three Lin-NOTCH repeats (LNR). The intracellular domain has the RBP-J-associated molecule (RAM) domain, six ankyrin repeats (ANK), nuclear localization sequences (NLS), a transactivation domain (TAD) required for activating transcription and a proline-, glutamate-, serine- and threonine-rich (PEST) domain which regulates NOTCH degradation. NOTCH1 and NOTCH2 are structurally similar, NOTCH3 and NOTCH4 are smaller proteins as they have fewer EGF repeats and lack TAD

NOTCH proteins are single pass transmembrane receptors that transduce extracellular signals into cells and mediate cell-cell interactions (Artavanis-Tsakonas et al. 1999). The mammalian NOTCH family consists of four NOTCH receptors (NOTCH1-4), which share extensive structural homology between its members (Fig. 1). Even across species as diverse as from flies to humans the NOTCH receptors are highly conserved (Schwanbeck and Just 2011). These receptors have distinct and partially overlapping functions. The extracellular domain (ECD) of NOTCH receptors consists of multiple Epidermal Growth Factor (EGF) like repeats necessary for ligand binding (Rebay et al. 1991). The intracellular domain (ICD) consists of the RBP-Jkappa-associated module domain (RAM), seven Ankyrin repeats, two nuclear localization signals, a transactivation domain and proline-glutamate-serine-threonine-rich (PEST) domain (Allman et al. 2002).

The RAM and the Ankyrin repeats are essential for signal transduction (Deregowski et al. 2006). The PEST domain consists of several phosphorylation sites, which regulate the NOTCH-ICD stability by ubiquitylation and are essential for NOTCH protein turn over (Andersson et al. 2011). The RAM domain is the primary binding site for the CSL {**C**p-binding factor 1 (CBF-1)/recombination signal sequence-binding protein-Jκ (RBP-Jκ), **S**uppressor of hairless [Su (h)], and **L**ag-1} (Iso et al. 2003) family of transcription factors, which modulate the transcription of NOTCH target genes like *Hairy and Enhancer of Split-1* (*HES1*) (Gordon et al. 2008).

There are five canonical NOTCH ligands in mammals belonging to Delta (Dll1, Dll3 and Dll4) and Jagged 1 (Jag 1 and Jag 2) families (D’Souza et al. 2008). Like the NOTCH receptors, these ligands are also single pass transmembrane proteins having multiple EGF-like repeats and cysteine rich sequences known as the Delta-Serrate-Lag2 (DSL) motif (Lubman et al. 2007). The EGF-like repeats and the DSL motifs on the ligands are required for them to bind and activate the NOTCH receptors on the neighbouring cells (Shimizu et al. 1999; Ohishi et al. 2003). These ligands have specificity for different NOTCH receptors. In murine B cell development, Dll1 functions as the main ligand for Notch2 whereas in T cell development Dll4 interacts with Notch1 (Mohtashami et al. 2010).

The posttranslational modifications of NOTCH receptors and NOTCH ligands are crucial in regulating NOTCH signalling. For example, a glycosyltransferase called Fringe modifies the EGF repeats of Notch receptors, which influences the proteolytic cleavage that release Notch-ICD (Moloney et al. 2000). The direct binding of Fringe to Notch receptors also regulates the specificity of Notch-ligand interactions (Okajima et al. 2003).

## NOTCH signalling pathway

**Fig. 2:**
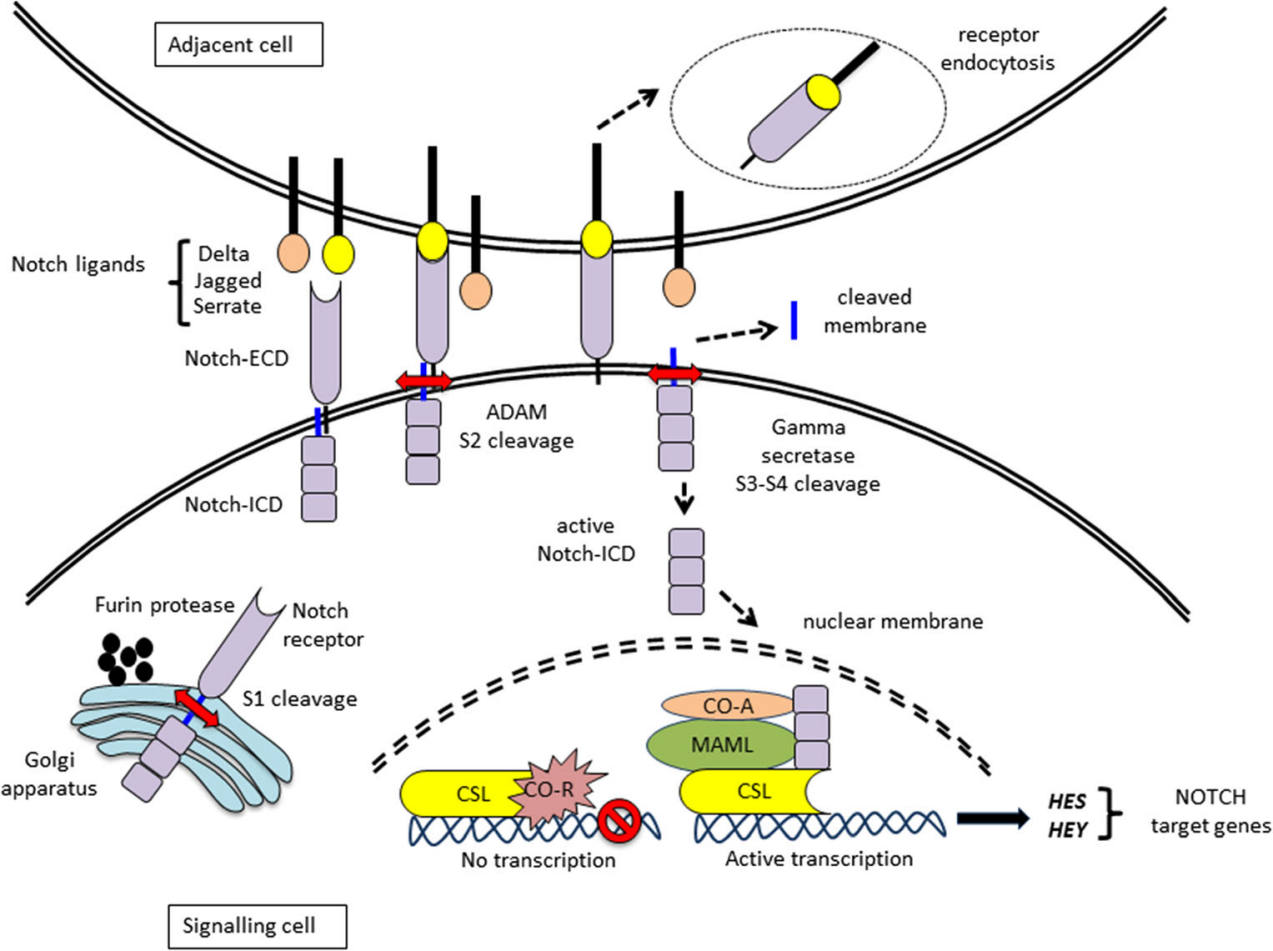
*NOTCH signaling pathway*. In the golgi apparatus the Notch receptor undergoes proteolytic processing, which is S1 cleavage mediated by Furin proteases. The receptor is transported to the cell surface membrane. The extracellular domain of the Notch receptor (Notch-ECD) in the signalling cell binds with the Notch ligands (Delta, Jagged, Serrate) expressed by the adjacent cell. This induces the second proteolytic step, S2 cleavage by ADAM metalloproteases, and leads to the endocytosis of the Notch-ECD into the ligand-expressing cell. This is followed by the release of the Notch intracellular domain (Notch-ICD) with a tethered membrane. This is now a substrate for the gamma secretase enzyme complex, which cleaves the Notch-ICD from the membrane by S3 and S4 cleavages. The resulting active Notch-ICD translocates to the nucleus and interacts with the CSL protein. In the absence of Notch-ICD, the CSL is bound to co-repressor. Binding of Notch-ICD with CSL results in the formation of an active complex with MAML and other co-activators and leads to the transcription of Notch targets *HES* and *HEY*

Within the cell, the NOTCH proteins undergo several proteolytic processes giving rise to many cleaved forms of NOTCH. Initially these receptors are cleaved by Furin proteases in the trans-Golgi (Logeat et al. 1998) (Fig. 2). This is termed as S1 cleavage, which forms heterodimers of NOTCH composed of NOTCH-ECD, a transmembrane and NOTCH-ICD. This structure spans the plasma membrane. The heterodimeric form of NOTCH is suggested to be the predominant form of NOTCH on the cell surface, however full length NOTCH has also been detected. The absence of S1 cleavage suppressed the formation of notches in the wings of *Drosophila* indicating a direct correlation of loss of S1 cleavage with loss of function of Notch (Lake et al. 2009).

The canonical NOTCH pathway is initiated by the binding of the ligands expressed on the surface of the neighbouring cells to the NOTCH-ECD. This results in the shedding of the NOTCH-ECD and its endocytosis into the ligand-expressing cell (Kopan and Ilagan 2009). The loss of the NOTCH-ECD exposes an S2 cleavage site for ADAM metalloproteases (Brou et al. 2000). Cleavage at the S2 site by ADAMs activates the receptor, which is still anchored to the plasma membrane. The γ-secretase enzyme complex (Presenilin 1 and 2, Nicastrin, APH1 and PEN2) cleaves the NOTCH receptor at S3 and S4 sites and subsequently releases the active NOTCH-ICD (De Strooper et al. 1999; Tien et al. 2009).

The two nuclear localization signals on the NOTCH-ICD direct its translocation to the nucleus. Here, the NOTCH-ICD binds to the CSL family of DNA binding proteins (RBP-J in mammals) (Tani et al. 2001). The binding of NOTCH-ICD removes the histone deacetylases (HDACs) and converts the co-repressor CSL into a co-activator. The CSL and NOTCH-ICD complex initiates transcription by recruiting chromatin remodelling proteins histone acetyl transferases (HATs) (Lai 2002). This finally results in the transcription of the prototypic NOTCH target gene *HES*, which functions as a major transcriptional repressor (Iso et al. 2003).

There are many other NOTCH target genes including *HEY* (subfamily of *HES*) (Monastirioti et al. 2010), c-MYC (Weng et al. 2006), CD25 (Adler et al. 2003), GATA-3 (Hozumi et al. 2008), cyclin D1 (Cohen et al. 2010), p21 (Guo et al. 2009), HOXA5, HOXA9 and HOXA10 (Weerkamp et al. 2006). The genes targeted by NOTCH either directly or indirectly are highly context specific.

The non-canonical pathway is CSL independent and can signal without conventional ligand interactions (Andersen et al. 2012). Some of the non-canonical NOTCH ligands include CCN3, microfibril-associated glycoprotein family (MAGP-1, MAGP-2), F3/Contactin 1, NB3/Contactin 6, which are involved in neural adhesion (D’Souza et al. 2008). One of the major effectors of the non-canonical NOTCH signaling is the Wnt/β-Catenin pathway (Hayward et al. 2005). Most of the non-canonical NOTCH interactions are observed in embryonic cells and in stem/progenitor cells, which implies the role of NOTCH signalling in undifferentiated cell populations (Andersen et al. 2012).

From this brief introduction it is evident that the field of NOTCH signaling is still young and much remains to be learnt about this complex pathway. Nevertheless, given the key role of NOTCH in co-operating with multiple signaling networks, particularly in stem cells, it is clear that modulation of this system may offer new therapeutic strategies in a variety of disease states. In this review we will focus on the development of such knowledge in blood stem cells and the application of this in Leukaemia.

## NOTCH in haematopoiesis

**Fig. 3:**
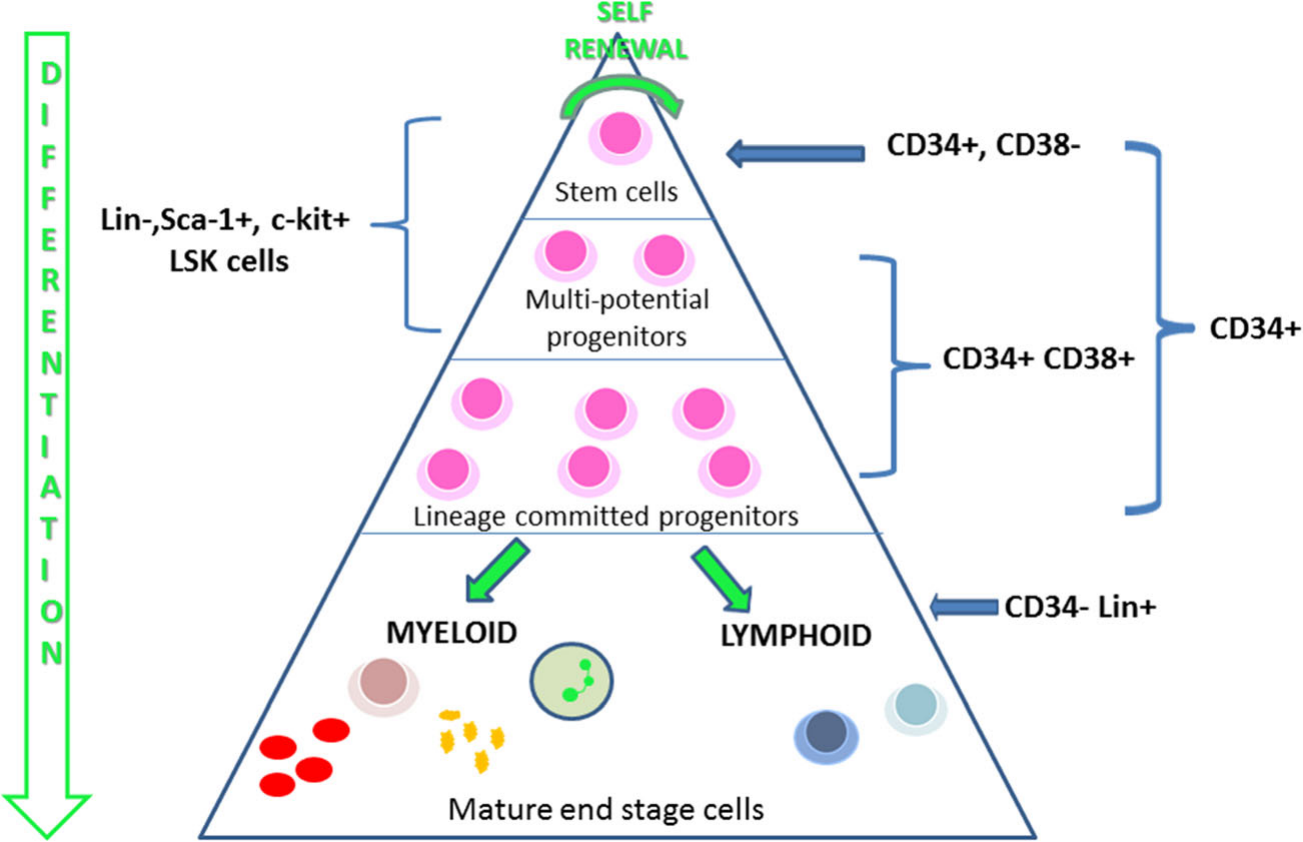
*Haematopoiesis.* Blood cell production is maintained by a small population of stem cells found in the bone marrow; these cells have the ability to self-renew or differentiate. Cells then differentiate to multi-potential and lineage committed progenitors; these cells can’t be distinguished morphologically, only by the use of clonogenic assays. The final stage of differentiation is when the cells develop their characteristic morphology and are released into the circulation

Blood cell production, haematopoiesis is the result of a tightly regulated balance of self-renewal and differentiation of haematopoietic stem cells (HSCs) (Fig. 3). During haematopoiesis NOTCH proteins are expressed at different stages of development. The activation of NOTCH in haematopoietic cells occurs during their interaction with other haematopoietic cells as well as with bone marrow stromal cells expressing NOTCH ligands (Bigas et al. 2010). Early studies have demonstrated the expression of NOTCH1 and NOTCH2 in the CD34^+^Lin^−^ precursors in the human bone marrow (Kojika and Griffin 2001; Ohishi et al. 2003). Stromal cells express NOTCH ligands Jagged1, Dll1 and Dll4 (Fernandez-Sanchez et al. 2011). Expression of all these ligands are observed in the thymus, which indicates the importance of NOTCH signaling in T cell development (de Pooter et al. 2006). Culturing murine **L**in^−^ **S**ca-1^+^c-**k**it^+^ (LSK) cells in the presence of immobilized Notch ligand Delta 1 reduced myeloid differentiation. When combined with IL-7, Notch signalling promoted early T- cell development whereas use of GM-CSF and Notch resulted in myeloid differentiation (Varnum-Finney et al. 2003). Under in vitro conditions endothelial cell production of NOTCH ligands was able to promote the self-renewal capacity of HSCs (Butler et al. 2010).

In the myeloid lineage, increased expression of NOTCH1 and NOTCH2 is reported in granulocytes (Ohishi et al. 2003). The expression of NOTCH1 decreases upon maturation of erythroid progenitors into erythroid cells (Walker et al. 2001). In Notch1 deficient mice, HSC generation was severely impaired with no HSC activity detected in the para-aortic splanchnopleura and in the yolk sac (Kumano et al. 2003). In mouse embryos, activation of Gata2 by Notch1/Rbp-j is essential for the onset of definitive haematopoiesis. For Notch1 to regulate Gata2 in the dorsal aorta, it has to be activated by Jagged-1 ligand, which suggests the importance of ligand-Notch specificity during haematopoiesis (Robert-Moreno et al. 2005). In transgenic mice, Notch1 signalling was active in HSCs and decreased upon their differentiation. The inhibition of Notch1 signalling enhanced the differentiation of HSCs in vitro but depleted the HSCs in vivo. It was shown in this study that Notch1 cooperates with Wnt signalling to maintain HSCs in undifferentiated state (Duncan et al. 2005). In contrast, another study that addressed the loss of function of canonical Notch signalling in murine adult HSCs ruled out any physiological roles for this pathway in these cells. However, this study did not address the effects of non-canonical NOTCH signalling on HSCs (Maillard et al. 2008). Therefore to fully understand the necessity of NOTCH signalling, it is essential to investigate the effects of non-canonical NOTCH signalling on HSCs.

The role of NOTCH signalling in haematopoiesis is complex. In murine LSK haematopoietic cells, constitutive Notch1 signalling (by forced expression of Notch1-ICD), gave rise to pluripotent, cytokine dependent HSCs. This study showed overexpression of active Notch1 having the ability to immortalize HSCs, which could lead to neoplasia (Varnum-Finney et al. 2000). Further, Notch1 activation inhibited the differentiation of murine Sca1^+^lin^−^ bone marrow cells, which maintained these cells in their ‘stemness’ rather than entering into the progenitor pool. Also, active Notch signalling in these cells determined their lineage commitment by favouring lymphopoiesis at the expense of myeloid cell production (Stier et al. 2002). In line with these observations, the retroviral expression of Hes1 in murine HSCs prolonged their self-renewal capacity ex vivo (Kunisato et al. 2003).

Notch ligands have promising clinical applications. Addition of Jagged-1 ligand to the human CD34^+^CD38^−^Lin^−^ cord blood cells expanded the progenitor cells and contributed to the short-term reconstitution in mice (Butler et al. 2010). Similar results were obtained with treating murine bone marrow LSK cells with Dll1 ligand (Delaney et al. 2005). Notch ligand Delta 1 was able to expand cord blood progenitors and when used in a clinical setting resulted in rapid haematopoietic recovery (Delaney et al. 2010). In these studies the haematopoietic stem/progenitor cells were exposed to high doses of Notch ligands resulting in enhanced Notch signalling, which could potentially decrease the effect induced by other haematopoietic signalling pathways that either co-operate or are independent of NOTCH signalling. Care should be taken in interpretation of in vitro experiments where cells are exposed to high NOTCH signalling as this does not represent its effects in steady state conditions. For example, Benveniste et al., found that human HSCs are expanded by NOTCH signaling in vitro but were not required for their maintenance or self-renewal in vivo (Benveniste et al. 2014).

Much of our understanding about NOTCH signalling in haematopoiesis comes from studies focused on the NOTCH1 receptor, the most potent of four receptors in activating transcription. Few studies have addressed the importance of other members of NOTCH family. In murine quiescent LSK cells, treatment with Dll1 or Jagged-1 activated Notch-2 and inhibited myeloid differentiation. Notch-2 activation modulated bone marrow stress recovery in mice receiving 5-Flurouracil by enhancing the rate of production of multi-potential progenitors with both short-term and long-term repopulating potential (Varnum-Finney et al. 2011). The expression of NOTCH4 in human bone marrow CD34^+^CD38^−^ primitive progenitor cells is high (along with high NOTCH1) compared to the mature progenitor fraction CD34^+^CD38^+^. Constitutively active NOTCH4 decreased monocytic and erythroid differentiation. Human cord blood cells with transduced NOTCH4-ICD showed higher engraftment potential in the bone marrow of immune-deficient mice. Active NOTCH4 favours differentiation in to lymphoid lineage similar to NOTCH1 (Vercauteren and Sutherland 2004).

## NOTCH in leukaemia

The constitutive activation of NOTCH signalling has been linked to excessive cell proliferation and arrested differentiation contributing to the development of cancer (Rosati et al. 2009). Given the importance of NOTCH signalling in haematopoiesis, it is not surprising that defective NOTCH signalling is involved in leukaemic transformation. Activating mutations of *NOTCH1* are observed in nearly 50 % of T-ALL and 30 % of Adult-T cell Leukaemia (ATL) patients (Weng et al. 2004). The mutations cause ligand independent activation of NOTCH1 receptors and enhanced stability of the NOTCH1-ICD. This subsequently leads to the increased proliferation and survival of leukaemic cells (Staal and Langerak 2008). A role for NOTCH3 in leukaemia was reported in studies using transgenic mice overexpressing NOTCH3-ICD, which gave rise to T cell leukaemia (Bellavia et al. 2002). In T-ALL, NOTCH3 has been shown to cooperate with MAPK pathway resulting in leukaemic survival (Masiero et al. 2011).

Gain of function mutations of *NOTCH1* have been reported in Chronic Lymphocytic Leukaemia (CLL) (Fabbri et al. 2011). In CLL, *NOTCH1* mutations impair FBW7 E3 ligase induced degradation of NOTCH1. These mutations were seen in patients with aggressive CLL refractory to chemotherapy (O’Neil et al. 2007). In CLL, *NOTCH1* mutations were suggestive of poor prognosis, but whether they are causative or drive the disease is not known (Rossi et al. 2012).

Activating *NOTCH2* mutations are observed in ~8 % of diffuse large B-cell lymphomas (Lee et al. 2009) and in 5 % of marginal zone lymphomas (Troen et al. 2008). The in vitro studies showed mutated NOTCH2 receptors having higher activity (Lee et al. 2009). There are currently no reports of NOTCH4 involvement in leukaemia.

## NOTCH in myeloid leukaemia

Unlike its established role in lymphoid leukaemias, there are conflicting reports regarding NOTCH signalling in myeloid leukaemia. This may reflect both disease heterogeneity and variation in experimental design. For example, the choice of experimental models differ between the research groups ranging from the use of immortalized leukaemic cell lines, primary cells from mice or humans and in vivo studies using leukaemic mouse models of different strains.

Several studies have attempted the expression profiling of Notch receptors and the Notch downstream target genes in myeloid leukaemias. One study reported AML patients having high *NOTCH1* gene expression in peripheral blood monocytes whereas *HES1* levels were low suggesting inactive NOTCH signalling (Chiaramonte et al. 2005). It is also to be noted that this group reported higher *JAGGED1* expression in AML samples than in T-ALL. Tohda et al. reported the expression of NOTCH1 receptor protein in 40 % of AML samples, but the activation of NOTCH1 targets was not assessed in this study (Tohda and Nara 2001). In a later study by the same group primary AML cells isolated from peripheral blood were treated with immobilized NOTCH ligands Jagged1 and Delta1; cellular responses to these ligands range from increased proliferation to suppressed proliferation (Tohda et al. 2005). Since the culture conditions were the same for all the samples, the reason for such differences could be the heterogeneity of the AML samples.

Whole genome microarray analysis of leukaemia initiating cells comprising the Lin^−^CD34^+^CD38^−^ and Lin^−^CD34^+^CD38^+^ from AML patients showed reduced levels of NOTCH target gene expression in comparison with the CD34^+^ bone marrow cells from normal donors. The CD34^+^ cells isolated from cord blood AML samples had less *HES1* mRNA expression. In these samples the *NOTCH1* levels were low but *NOTCH2* mRNA levels were surprisingly high. The authors also report decreased *NOTCH1* and NOTCH targets and increased *NOTCH2* in the leukaemia initiating HSC population in an MLL-AF9 AML mouse model. Inducible activation of NOTCH1-ICD suppressed the disease phenotype and was associated with a decrease in the leukaemic initiating cell population. Similarly the MLL-AF9 AML mice with induction of NOTCH2-ICD survived while the control leukaemic mice did not. Treatment of primary AML cells with Notch ligand Dll4 induced differentiation to a macrophage lineage followed by apoptotic clearance (Lobry et al. 2013). In another microarray gene expression profiling study CD34+ bone marrow cells from AML patients had *NOTCH1* and *NOTCH2* expression similar to the normal donor samples, however the levels of NOTCH targets *HES1* and *DELTEX1* were lower in AML cells indicating reduced NOTCH signalling despite the expression of NOTCH receptors (Kannan et al. 2013). The corresponding protein expression analysis revealed NOTCH2 expressed at higher level, which was in agreement with the report of Lobry et al. (Lobry et al. 2013). Activation of the NOTCH signalling pathway by induced expression of the NOTCH1-ICD inhibited the proliferation of several AML cell lines and was attributed to the associated increase in HES1 whereas inhibition of the overall NOTCH signalling had no effect on these cell lines. An interesting observation made in this report is that the active intracellular domains for each of the four NOTCH receptors were anti-proliferative with varying effect indicating that the growth inhibition is most likely mediated by a downstream component common to the NOTCH receptors. Accordingly, it was found that HES1 was induced by all NOTCH receptors and its activation was sufficient to induce growth arrest, which was accompanied by an increase in p53 and reduced anti-apoptotic BCL2 protein. Treatment of human primary AML cells with a NOTCH agonist peptide induced significant apoptosis (Kannan et al. 2013). These findings expand our understanding about the relative contributions of the four NOTCH receptors and the downstream signals in myeloid leukaemia and should pave the way for experiments designed to take these findings to clinical applications.

Activating mutations of *NOTCH1* have been postulated to be restricted to T-ALL and are rare in myeloid leukaemias (Palomero et al. 2006). However, a recent genome wide alternate splicing study reported *NOTCH2* along with *FLT3* as the most aberrantly spliced genes detected in the majority of the AML patients (Adamia et al. 2014). The NOTCH2 variants had a specific pattern of expression during the different stages of AML with 79 % AML patients expressing the variant *NOTCH2-Va,* during remission 40 % of AML patients were negative for *NOTCH2-Va* and during relapse 80 % of AML patients expressed this variant. High *NOTCH2-Va* expression was also associated with poor clinical outcome in the intermediate cytogenetic risk AML groups. The CD34^+^ bone marrow cells of AML patients with high expression of *NOTCH2* splice variant had decreased mRNA expression of *NOTCH* targets *HES1*, *HEY1* and *DTX1* compared with CD34^+^ AML cells expressing full length *NOTCH2*. This suggests that the expression of *NOTCH2* splice variants have oncogenic potential and results in the inactivation of NOTCH pathway and this process might be contributing to AML pathology (Adamia et al. 2014). In contrast with the above observations, there have been reports of elevated expression of *NOTCH1*, *Dll4* and *HES1* transcripts and corresponding increase in NOTCH1-ICD, Dll4 and HES1 protein in AML patients. Also, AML patients with highest NOTCH1 expression were reported to have poor overall survival (Zhang et al. 2013).

In Chronic Myeloid Leukaemia (CML), inhibition of NOTCH1 expression in K562 cells induced erythroid maturation of these cells (Lam et al. 2000). K562 cells have been reported to be resistant to NOTCH1 inhibition with gamma secretase inhibitor (GSI) (Moellering et al. 2009; Ren and Cowell 2011). However, when K562 cells were induced to express the non-canonical NOTCH ligand CCN3, they were responsive to GSI inhibition, which resulted in reduced colony formation. Besides K562 cells, other CML cell line models like KCL22 and LAMA were sensitive to GSI when used in combination with either recombinant CCN3 or imatinib. BCR-ABL knockdown in all these cell lines reduced NOTCH1 signalling and inhibition of NOTCH was anti-proliferative suggesting NOTCH1 to have an oncogenic role in CML (Suresh et al. 2013). Similarly, in a transgenic CML mouse model overexpressing Bcr-Abl, activation of Notch1 was observed. In this model, activated Notch1-ICD was shown to cooperate with Bcr-Abl contributing to CML blast crisis (Mizuno et al. 2008). Contrary to these observations, inactivation of Notch1 in mouse HSCs induced chronic myelomonocytic leukaemia (CMML) and Notch1 here has been suggested to have tumour suppressor roles (Klinakis et al. 2011). In this study, conditional knockdown of Notch1/2/3 genes in mouse models induced CMML like disease and introduction of Notch1 or Notch2 could rescue the disease phenotype. This study also reported Notch1-ICD expression in murine leukaemia initiating cells upregulating HES1 levels, which led to the repression of myeloid expansion. However, HES1 has been previously reported to cause CML blast crisis transformation in mice (Nakahara et al. 2010). These different outcomes of defective NOTCH1 signalling across myeloid leukaemias show the complexity of this signalling pathway. This reflects the problem of generalizing about NOTCH1 signalling in haematopoiesis.

## Conclusion

There is substantial evidence establishing the role of NOTCH in haematopoiesis and leukaemia. Since constitutively active NOTCH-ICD contributes to cancer progression in many cases, several small molecule inhibitors that either block the function of gamma secretase complex or monoclonal antibodies to NOTCH receptors/ligands are being developed and many of these compounds are currently in clinical trials. In laboratory settings GSI has been widely used to investigate the effects of NOTCH inhibition. However, the first Phase I clinical trial for the GSI MK-0752 (developed by Merck, Whitehouse Station, NJ, USA) for relapsed or refractory T-ALL patients and advanced breast cancers was met with severe toxicity (Takebe et al. 2014). GSI has a very broad spectrum of action, it inhibits signalling of all four NOTCH receptors and it has other substrates like CD44 (Murakami et al. 2003), ERBB4 (Vidal et al. 2005) and Cadherins (Marambaud et al. 2002). Monoclonal antibodies to NOTCH ligands (Tran et al. 2013) and receptors are under development to improve target specificity (Wu et al. 2010). Phase I studies with demcizumab; a humanized antibody to Dll4 (OMP-21M18, OncoMed Pharmaceuticals, CA, USA) and OMP-59R5 and OPM-52M51 (monoclonal antibodies to NOTCH 2 and NOTCH 3 respectively) are currently underway (Takebe et al. 2014).

The role of NOTCH as a potential target for cancer therapy is a new field of research, as the initial observation of its importance in cancer was known only in 2004 (Weng et al. 2004). As discussed in this review, the significance of NOTCH in myeloid leukaemias is not well defined unlike its oncogenic potential in T-ALL. Therefore it is too early to know how the NOTCH signalling pathway could be therapeutically useful in myeloid leukaemias. Currently, targeting NOTCH has tremendous potential in fine-tuning a signalling pathway that cooperates with multiple signalling networks. However, leukaemias being stem cell disorders, it is essential to understand how NOTCH inhibition affects normal stem cells while investigating NOTCH based anticancer therapies.
